# Os Melhores Artigos de 2022 nos Arquivos Brasileiros de Cardiologia e na Revista Portuguesa de Cardiologia

**DOI:** 10.36660/abc.20230342

**Published:** 2023-07-19

**Authors:** Gláucia Maria Moraes de Oliveira, Ricardo Fontes-Carvalho, Nuno Cardim, Carlos Eduardo Rochitte

**Affiliations:** 1 Faculdade de Medicina Universidade Federal do Rio de Janeiro Rio de Janeiro RJ Brasil Faculdade de Medicina – Universidade Federal do Rio de Janeiro, Rio de Janeiro, RJ – Brasil; 2 Instituto do Coração Edson Saad Universidade Federal do Rio de Janeiro Rio de Janeiro RJ Brasil Instituto do Coração Edson Saad – Universidade Federal do Rio de Janeiro, Rio de Janeiro, RJ – Brasil; 3 Departamento de Cardiologia Centro Hospitalar de Vila Nova de Gaia/Espinho Espinho Portugal Departamento de Cardiologia – Centro Hospitalar de Vila Nova de Gaia/Espinho, Vila Nova de Gaia/Espinho – Portugal; 4 UniC Faculdade de Medicina Universidade do Porto Porto Portugal Cardiovascular Research Center (UniC) - Faculdade de Medicina - Universidade do Porto, Porto – Portugal; 5 Serviço de Cardiologia Hospital CUF Descobertas Lisboa Portugal Serviço de Cardiologia Hospital CUF Descobertas, Lisboa – Portugal; 6 Nova Medical School Universidade Nova Lisboa Portugal Nova Medical School, Universidade Nova, Lisboa – Portugal; 7 Instituto do Coração Faculdade de Medicina Universidade de São Paulo São Paulo SP Brasil Instituto do Coração (InCor) – Faculdade de Medicina da Universidade de São Paulo, São Paulo, SP – Brasil; 8 Hospital do Coração São Paulo SP Brasil Hospital do Coração (HCOR), São Paulo, SP – Brasil

**Keywords:** Brasil, Cooperação Técnica/tendências, Disseminação da Informação, Doenças Cardiovasculares, Fator de Impacto

## Introdução

Dando seguimento à tradição dos últimos anos,^1 , 2^ a Revista Portuguesa de Cardiologia e os Arquivos Brasileiros de Cardiologia voltam este ano a publicar um artigo especial com a seleção dos 10 melhores artigos originais publicados em cada uma dessas revistas ( Tabelas 1 e 2 ).

**Tabela 1:** – Lista com a seleção dos 10 melhores artigos publicados nos Arquivos Brasileiros de Cardiologia em 2022

Autores	Título do artigo, DOI e *link* para acesso
Zarife et al. ^4^	Variabilidade da pressão arterial em única visita e risco cardiovascular em participantes do ELSA-Brasil DOI: 10.36660/abc.20210804 https://abccardiol.org/article/variabilidade-da-pressao-arterial-em-unica-visita-e-risco-cardiovascular-em-participantes-do-elsa-brasil/
Porto et al. ^5^	Efeitos agudos da bebida energética sobre parâmetros autonômicos e cardiovasculares em indivíduos com diferentes capacidades cardiorrespiratórias: um ensaio controlado, randomizado, crossover e duplo cego DOI: 10.36660/abc.20210625 https://abccardiol.org/article/efeitos-agudos-da-bebida-energetica-sobre-parametros-autonomicos-e-cardiovasculares-em-individuos-com-diferentes-capacidades-cardiorrespiratorias-um-ensaio-controlado-randomizado-crossover-e-duplo/
Jannes et al. ^6^	Rastreamento para hipercolesterolemia familiar em pequenos municípios: a experiência do programa HipercolBrasil em 11 municípios brasileiros DOI: 10.36660/abc.20201371 https://abccardiol.org/article/rastreamento-para-hipercolesterolemia-familiar-em-pequenos-municipios-a-experiencia-do-programa-hipercolbrasil-em-11-municipios-brasileiros/
Kravchychyn et al. ^7^	O papel dos níveis séricos de ANP na perda de peso, risco cardiometabólico e composição corporal de adolescentes com obesidade submetidos a terapia interdisciplinar DOI: 10.36660/abc.20200735 https://abccardiol.org/article/o-papel-dos-niveis-sericos-de-anp-na-perda-de-peso-risco-cardiometabolico-e-composicao-corporal-de-adolescentes-com-obesidade-submetidos-a-terapia-interdisciplinar/
Vieira et al. ^8^	Avaliação do impacto da implantação de um sistema de ambulância pré-hospitalar sobre mortalidade por infarto agudo do miocárdio em um país em desenvolvimento DOI: 10.36660/abc.20210953 https://abccardiol.org/article/avaliacao-do-impacto-da-implantacao-de-um-sistema-de-ambulancia-pre-hospitalar-sobre-mortalidade-por-infarto-agudo-do-miocardio-em-um-pais-em-desenvolvimento/
Bianco et al. ^9^	Estratégia fármaco-invasiva no infarto do miocárdio: análise descritiva, apresentação de sintomas isquêmicos e preditores de mortalidade https://abccardiol.org/article/estrategia-farmaco-invasiva-no-infarto-do-miocardio-analise-descritiva-apresentacao-de-sintomas-isquemicos-e-preditores-de-mortalidade/
Cerci et al. ^10^	O impacto da COVID-19 no diagnóstico de doenças cardíacas na américa latina uma subanálise do INCAPS COVID DOI: 10.36660/abc.20210388 https://abccardiol.org/article/o-impacto-da-covid-19-no-diagnostico-de-doencas-cardiacas-na-america-latina-uma-subanalise-do-incaps-covid/
Forno et al. ^11^	Estimulação do ramo esquerdo do sistema His-Purkinje: experiência inicial DOI: 10.36660/abc.20201085 https://abccardiol.org/article/estimulacao-do-ramo-esquerdo-do-sistema-his-purkinje-experiencia-inicial/
Fernández-Rodríguez et al. ^12^	Ajustando a RFR por preditores de discordância, “A RFR Ajustada”: uma metodologia alternativa para melhorar a capacidade diagnóstica dos índices coronarianos DOI: 10.36660/abc.20220176 https://abccardiol.org/article/ajustando-a-rfr-por-preditores-de-discordancia-a-rfr-ajustada-uma-metodologia-alternativa-para-melhorar-a-capacidade-diagnostica-dos-indices-coronarianos/
Soares et al. ^13^	O treinamento físico resistido atenua as disfunções ventriculares esquerdas em modelo de hipertensão arterial pulmonar DOI: 10.36660/abc.20210681 https://abccardiol.org/article/o-treinamento-fisico-resistido-atenua-as-disfuncoes-ventriculares-esquerdas-em-modelo-de-hipertensao-arterial-pulmonar/

**Tabela 2:** – Lista com a seleção dos 10 melhores artigos publicados na Revista Portuguesa de Cardiologia em 2022

Autores	Título do artigo, DOI e *link* para acesso
Gavina et al. ^14^	Prognostic implications of fibrosis in low-risk aortic stenosis patients DOI: 10.1016/j.repc.2021.02.017 https://www.revportcardiol.org/pt-prognostic-implications-fibrosis-in-low-articulo-S0870255121004546
Pereira et al. ^15^	Stent-Save a Life international survey on the practice of primary percutaneous coronary intervention during the COVID-19 pandemic DOI: 10.1016/j.repc.2021.04.006 https://www.revportcardiol.org/pt-stent-save-life-international-survey-on-articulo-S0870255121005217
Pinto et al. ^16^	Digital home-based multidisciplinary cardiac rehabilitation: how to counteract physical inactivity during the COVID-19 pandemic DOI: 10.1016/j.repc.2021.05.013 https://www.revportcardiol.org/pt-digital-home-based-multidisciplinary-cardiac-rehabilitation-articulo-S0870255121004637
Almeida et al. ^17^	Prognostic value of brain natriuretic peptide in ST-elevation myocardial infarction patients: a Portuguese registry DOI: 10.1016/j.repc.2020.12.016 https://www.revportcardiol.org/pt-prognostic-value-brain-natriuretic-peptide-articulo-S0870255121004431
Cai et al. ^18^	High expression of SGK1 in thrombosis of acute ST-segment elevation myocardial infarction: based on proteomics analysis of intracoronary thrombosis DOI: 10.1016/j.repc.2021.02.023 https://www.revportcardiol.org/pt-high-expression-sgk1-in-thrombosis-articulo-S0870255122000233
Apolinário et al. ^19^	Infective endocarditis: Epidemiology and prognosis DOI: 10.1016/j.repc.2021.02.027 https://www.revportcardiol.org/pt-infective-endocarditis-epidemiology-prognosis-articulo-S0870255122000361
Menezes et al. ^20^	Development of deep learning segmentation models for coronary X-ray angiography: quality assessment by a new global segmentation score and comparison with human performance DOI: 10.1016/j.repc.2022.04.001 https://www.revportcardiol.org/pt-development-deep-learning-segmentation-models-articulo-S0870255122001354
Abreu et al. ^21^	Mechanical circulatory support in children: strategies, challenges and future directions DOI: 10.1016/j.repc.2021.03.011 https://www.revportcardiol.org/pt-mechanical-circulatory-support-in-children-articulo-S0870255121005230
Lanzas et al. ^22^	Training program in resuscitation maneuvers delivered by teachers in a school setting: an economic argument DOI: 10.1016/j.repc.2021.02.015 https://www.revportcardiol.org/pt-training-program-in-resuscitation-maneuvers-articulo-S0870255121003772
Hormigo et al. ^23^	Protocol-based cardiotoxicity monitoring in hydroxychloroquine medicated COVID-19 pediatric patients DOI: 10.1016/j.repc.2021.01.018 https://www.revportcardiol.org/pt-protocol-based-cardiotoxicity-monitoring-in-hydroxychloroquine-articulo-S0870255121004297

Esta seleção, efetuada pelos corpos editoriais dos dois periódicos, foi difícil e complexa, uma vez que não foi possível incluir muitos artigos de excelente qualidade científica, mas ilustra o grande dinamismo científico da investigação cardiovascular (básica, clínica e de translação) que é efetuada por esses países de língua portuguesa.

Essa união de esforços para partilhar o que foi melhor produzido em Cardiologia nos países de língua portuguesa (PLP) se reveste de grande importância, especialmente pela redução expressiva das taxas de mortalidade cardiovascular padronizadas por idade atribuíveis aos fatores de risco (FR), notadamente nos PLP com melhores índices socioeconômicos, como Brasil e Portugal, entre 1990 e 2019,^3^ sendo importante compartilhar experiências exitosas documentadas nos estudos desses dois países, de onde se originam os respectivos periódicos.

### Fatores de risco cardiovascular

Observou-se importância crescente dos FR dietéticos e metabólicos, em paralelo com redução de taxas de tabagismo nos PLP e correlação negativa entre a variação das taxas de mortalidade por doenças cardiovasculares (DCV) atribuíveis aos FR e o índice sociodemográfico (SDI).^3^ É importante mencionar que as taxas de mortalidade por DCV atribuível à pressão arterial sistólica (PAS) elevada permaneceram no primeiro lugar do *ranking* em todos os PLP entre 1990 e 2019, com maiores reduções observadas em Portugal e no Brasil.

Um estudo transversal foi conduzido (2008-2010) com dados basais de 14.357 participantes do ELSA-Brasil, sem história de DCV, com o objetivo de avaliar a associação entre a variabilidade da pressão arterial (VPA), aferida em uma única visita, e o risco cardiovascular. A VPA foi quantificada pelo coeficiente de variação de três medidas padronizadas da PAS realizadas com um oscilômetro. Medidas antropométricas e exames laboratoriais também foram realizados. O risco cardiovascular foi avaliado pelo estimador de risco de doença cardiovascular aterosclerótica empregando análise de regressão logística multivariada, com nível de significância de 5%. Os autores observaram que o risco cardiovascular significativamente maior foi associado com VPA elevada para ambos os sexos. Uma prevalência significativamente maior de alto risco foi observada mais em homens que em mulheres em todos os quartis, com a maior diferença observada no quarto quartil de variabilidade.^4^ Ações conjuntas, como a implementação da diretriz para manejo da hipertensão arterial em cuidados primários nos PLP, que visem à efetivação de medidas de prevenção primária poderão reduzir os desfechos relacionados com a doença hipertensiva, especialmente acidente vascular cerebral e infarto agudo do miocárdio (IAM), as principais causas de mortalidade nos PLP.^24^

Além da elevação da PAS, os FR dietéticos e metabólicos justificaram uma maior variação da carga de DCV, correlacionada com o SDI nos PLP.^3^ Um maior consumo de alimentos ultraprocessados (UP) foi associado a maior risco de incidência e mortalidade por DCV, sugerindo que os alimentos UP devam ser banidos da dieta ou minimamente consumidos.^25^ Entre os alimentos UP, as bebidas energéticas (BEs) são amplamente consumidas no meio esportivo para melhorar o desempenho aeróbico, mas os efeitos agudos sobre a fisiologia cardiovascular são poucos conhecidos. Porto et al.^5^ avaliaram o efeito agudo de uma BE (250 mL) com valor energético de 45 kcal (composto por 11,2 g de carboidratos, 80 mg de sódio, 32 mg de cafeína, 400 mg de taurina, 4,6 mg de niacina, 2 mg de ácido pantotênico, 0,5 mg de vitamina B6, 0,4 mg de vitamina B12, 240 mg de glucoronolactona e 20 mg de inositol) sobre a variabilidade da frequência cardíaca e a recuperação cardiovascular após exercício aeróbico moderado precedido pelo consumo da BE. O estudo foi randomizado, duplo-cego, cruzado, controlado por placebo e feito com 28 jovens adultos divididos em dois grupos de acordo com o pico de consumo de oxigênio (pico de VO2): (1) pico de VO2 alto (AO) – pico de VO2 > 52,15 mL/kg/min; e (2) pico de VO2 baixo (BO) – pico de VO2 < 52,15 mL/kg/min. Os autores concluíram que a ingestão aguda de BE não teve efeito sobre a PAS e pressão diastólica, a saturação arterial de oxigênio por oximetria de pulso (SpO2) e a taxa respiratória, mas atrasou a recuperação da frequência cardíaca após o exercício em indivíduos com baixa ou alta capacidade cardiorrespiratória. Os autores ainda chamaram a atenção para que os indivíduos com doenças cardiovasculares e metabólicas evitem o uso de BE (como um suplemento) antes da prática de exercício físico.

Os riscos dietéticos, a glicemia de jejum elevada, o colesterol de lipoproteína de baixa densidade (LDL-C) elevado e a poluição do ar estiveram entre os cinco FR mais importantes na maioria dos PLP em 1990 e em 2019, e houve uma tendência à correlação inversa entre o SDI e o percentual de mudança, com significância estatística para os riscos dietéticos, LDL-C elevado e PAS elevada nos PLP, sendo importante fazer o rastreamento desses FR.^3^ Níveis sanguíneos elevados de LDL-C ocorrem na hipercolesterolemia familiar (HF), uma doença autossômica dominante associada à ocorrência de DCV aterosclerótica precoce. O HipercolBrasil é um programa de rastreamento em cascata para HF que já identificou mais de 2.000 indivíduos com variantes genéticas causadoras de HF, através do rastreamento em cascata de casos índices referidos, indivíduos com hipercolesterolemia e suspeita clínica de HF. Um estudo que realizou o rastreamento em cascata direcionado a 11 pequenos municípios brasileiros com suspeita de alta prevalência de indivíduos com HF (achado prévio de indivíduos com o fenótipo homozigoto no mesmo município ou em regiões com alta frequência de IAM) identificou 105 casos índices e 409 familiares de primeiro grau com rendimento de 4,67 familiares por caso índice. Os autores sugeriram que regiões geográficas específicas com suspeita de alta prevalência de HF justificariam uma abordagem em cascata direcionada para a identificação de aglomerações de indivíduos com HF.^6^

A obesidade, caracterizada pelo aumento do índice de massa corporal (IMC), foi o sexto FR mais importantes na maioria dos PLP em 1990 e em 2019.^3^ É importante ressaltar que mais de 1 bilhão de pessoas no mundo são obesas – 650 milhões de adultos, 340 milhões de adolescentes e 39 milhões de crianças. A Organização Mundial da Saúde (OMS) estima que, até 2025, aproximadamente 167 milhões de pessoas – adultos e crianças – se tornarão menos saudáveis por estarem com sobrepeso ou obesidade.^26^ Kravchychyn et al.^7^ estudaram a hipótese de que a terapia clínica interdisciplinar para perda de peso poderia melhorar a prevalência da síndrome metabólica (Smet) e os riscos cardiometabólicos em adolescentes com obesidade e que essa melhora seria associada a mudanças nos níveis de peptídeo natriurético atrial (ANP). Os autores avaliaram 73 adolescentes com obesidade submetidos a terapia interdisciplinar para perda de peso de 20 semanas, incluindo abordagem clínica, nutricional, psicológica e de exercícios físicos. A composição corporal, as análises bioquímicas e a pressão sanguínea foram também medidas. Após o tratamento, os voluntários foram divididos de acordo com os níveis de plasma do ANP aumentado (n = 31) ou ANP reduzido (n = 19). Observou-se redução significativa na gordura corporal, na razão de triglicerídeos/colesterol de lipoproteína de alta densidade (TG/HDL-c) e na prevalência de Smet (de 23% para 6%) somente no grupo com ANP aumentado, ainda que ambos os grupos tivessem reduções significativas de peso corporal, do IMC e das circunferências de cintura, pescoço e quadril e aumento da massa livre de gordura.

### Doença cardíaca isquêmica

A procura por novos marcadores de prognóstico em pessoas com síndrome coronária aguda (SCA) continua. Em um estudo proveniente do Registo Nacional de SCA da Sociedade Portuguesa de Cardiologia, em que participaram dezenas de centros de Portugal, os autores pretenderam avaliar o impacto prognóstico dos valores de peptídeo natriurético cerebral (BNP) durante a hospitalização por SCA com supradesnivelamento do segmento ST.^17^ Foram avaliados dados de 1.650 pacientes, sendo que cerca de 21% tinham valores elevados de BNP. Tal como seria de esperar, observou-se que as pessoas com valores mais elevados de BNP eram mais idosas, tinham mais comorbilidades, menor fração de ejeção e maior gravidade de doença coronária. Observou-se ainda que, mesmo após a utilização de técnicas de *propensity matching score* , a presença de valores mais elevados de BNP (> 400 pg/mL) foi um importante marcador de prognóstico, quer de mortalidade intra-hospitalar, quer de mortalidade ao fim de 1 ano. Esse estudo sugere que a dosagem dos valores de BNP é uma ferramenta simples e facilmente acessível para a estratificação adicional de risco em pacientes admitidos com SCA com supradesnivelamento do segmento ST.

Na fisiopatologia da SCA, a trombose desempenha um papel essencial no desenvolvimento do evento, sendo importante continuar a explorar os mecanismos fisiopatológicos e as vias envolvidas no desencadear do processo de trombose intracoronário. Em um estudo publicado na Revista Portuguesa de Cardiologia, Cai et al.^18^ analisaram a expressão proteômica de trombos intracoronários extraídos por trombectomia aspirativa de pacientes com SCA com supradesnivelamento do segmento ST (n = 30). Esse estudo é potencialmente relevante porque estudos prévios demonstraram que a análise proteômica pode ser útil na identificação de novos biomarcadores e novos alvos terapêuticos em várias doenças. Nesse estudo, observou-se um aumento significativo da expressão da proteína SGK1 no trombo dos pacientes comparativamente ao grupo controle (17,21±2,36 *versus* 4,14±1,17%, p < 0,05). A cinase 1 induzida pelo soro/glicocorticoide (SGK1) é um efetor da via de sinalização do fosfatidilinositol-3’-cinase (PI3K), podendo representar um novo alvo terapêutico na prevenção de eventos aterotrombóticos, sendo necessários mais estudos para confirmar essa hipótese.

O manejo efetivo dos pacientes com IAM está diretamente ligado ao tempo para assistência médica, e aproximadamente metade dos óbitos atribuídos a IAM resultam de parada cardíaca fora do hospital, reforçando a importância do atendimento pré-hospitalar e do desenvolvimento de sistemas de atenção para IAM baseados em evidências. Vieira et al.^8^ analisaram os impactos da implantação do atendimento pré-hospitalar nas taxas de mortalidade geral e intra-hospitalar por IAM e na taxa de internação por IAM em 853 municípios de Minas Gerais, de 2008 a 2016, empregando o modelo hierárquico de Poisson. A implantação do Serviço de Atendimento Móvel de Urgência (SAMU) foi associada à diminuição da mortalidade por IAM (razão de chances [OR, de *odds ratio* ] = 0,967, intervalo de confiança de 95% [IC95%] 0,936 a 0,998) e da mortalidade intra-hospitalar por IAM (OR = 0,914, IC95% 0,845 a 0,986), sem associação significativa com internações (OR = 1,003, IC95% 0,927 a 1,083). Os autores concluiram que esses achados reforçam o papel fundamental do cuidado pré-hospitalar no cuidado do IAM e a necessidade de investimentos nesse serviço para melhorar os desfechos clínicos em países de baixa e média renda.

Durante o processo diagnóstico clínico e eletrocardiográfico do IAM, podem surgir diferenças em relação a como os sintomas são tratados, especialmente em subgrupos específicos, como mulheres ou pessoas mais idosas. Um estudo com 2.290 pacientes reportou que as mulheres apresentaram alta prevalência de sintomas atípicos; maior tempo entre o início dos sintomas e a procura por atendimento; e atraso entre a chegada ao pronto-socorro e a fibrinólise. A mortalidade hospitalar foi de 5,6%. As taxas de mortalidade hospitalar eram mais altas em mulheres, em pacientes com diabetes melito, obesidade, doença renal crônica e acidentes vasculares prévios e em idosos. A disparidade relacionada a sexo persiste nas mulheres, com demoras no reconhecimento dos sintomas de isquemia e no início imediato de terapia fibrinolítica, levando a piores resultados clínicos. Os autores salientaram que a aplicabilidade do escore de Killip-Kimball para prever eventos fatais com precisão deve ser destacada, independentemente da apresentação clínica do evento isquêmico agudo, medido na primeira consulta médica, especialmente na estratégia fármaco-invasiva.^9^

### Covid-19 e doença cardiovascular

A pandemia pelo coronavírus 2 da síndrome respiratória aguda grave (SARS-CoV-2) teve um enorme impacto nos sistemas de saúde em todo o mundo, nomeadamente nos cuidados prestados aos pacientes com doença cardiovascular. Durante o ano de 2022, foram publicados vários artigos que mostraram o impacto que a covid-19 teve na doença cardiovascular. Apesar de, atualmente, ser clara a enorme disrupção que a covid-19 teve nos cuidados prestados aos pacientes cardiovasculares, falta ainda estudar e determinar os impactos que terá a médio e longo prazo.

A International Atomic Energy Agency realizou uma pesquisa mundial avaliando mudanças nos volumes diagnósticos cardíacos decorrentes da covid-19 entre março e abril de 2020 e comparados com março de 2019. Foram coletados dados de distanciamento social a partir dos Relatórios de Mobilidade da Comunidade da empresa Google e a incidência de covid-19 por país a partir de Our World in Data. Os autores analisaram 194 centros que realizam procedimentos diagnósticos cardíacos em 19 países da América Latina e observaram que, em comparação com o mês de março de 2019, os volumes dos procedimentos diagnósticos cardíacos diminuíram 36% em março de 2020 e 82% em abril de 2020. As maiores reduções ocorreram em relação aos testes de estresse ecocardiográfico (91%), testes ergométricos de esteira (88%) e escore de cálcio por tomografia computadorizada (87%), com pequenas variações entre as sub-regiões da América Latina. As mudanças em padrões de distanciamento social (p < 0,001) estavam mais fortemente associadas com a redução do volume do que a incidência de covid-19 (p = 0,003).^10^

Em outro artigo multicêntrico publicado na Revista Portuguesa de Cardiologia, os autores pretenderam avaliar o impacto da covid-19 na admissão de pacientes com infarto do miocárdio.^15^ Nesse estudo, foram analisados os dados de 17 países incluídos no projeto “Stent Save a Life”. Foi possível observar que, nos 2 primeiros meses da pandemia, houve uma redução global de 27,5% nas admissões hospitalares por infarto do miocárdio e uma redução de 20% das admissões por infarto do miocárdio com supradesnivelamento do segmento ST. É interessante ressaltar que essa redução foi observada em todos os países, exceto no Egito e na Rússia, porque foram países em que a pandemia teve um atingimento mais tardio.

Esses resultados são semelhantes aos observados em dois outros estudos que analisaram em maior detalhe a realidade portuguesa. No primeiro estudo, Oliveira et al.^27^ avaliaram retrospectivamente o impacto da covid-19 em dois centros portugueses, tendo demonstrado uma redução de 26% do número de infarto com supradenivelamento do segmento ST. Observou-se tendência para um aumento dos tempos de atraso do sistema e aumento do número de complicações mecânicas, com consequente aumento da mortalidade dos pacientes (1,9% *versus* 12,1%). Esses dados são muito significativos e devem suscitar uma reflexão sobre os efeitos indiretos da pandemia por covid-19,^28^ mas também devem obrigar a preparar melhor a organização dos sistemas de saúde para eventuais novas pandemias. Esses resultados são semelhantes aos de outro estudo publicado em 2022,^29^ que também avaliou o impacto da covid-19 em um dos maiores hospitais do Norte de Portugal. Observou-se uma redução global das admissões hospitalares por infarto do miocárdio e geralmente casos mais graves com maior disfunção ventricular esquerda à data da alta (55% *versus* 39%).

Contudo, a pandemia por covid-19 não trouxe apenas aspectos negativos; obrigou também a uma adaptação dos profissionais e dos sistemas de saúde para melhorar a assistência aos pacientes no âmbito da telemedicina. Em um artigo publicado por Pinto et al.,^16^ os autores analisaram o impacto de um programa de reabilitação cardíaca a distância (por via digital) desenvolvido durante a pandemia de covid-19, que inclui consultas a distância, sessões de grupo de exercício e educação em saúde e psicológica. Foram incluídas nesse programa 95 pessoas com doença cardiovascular, tendo sido demonstrado que esse programa de reabilitação cardíaca a distância aumentava o tempo de atividade física dos pacientes e diminuía os níveis de sedentarismo. Foi demonstrado que a utilização desses programas de reabilitação cardíaca a distância foi segura e podem ser utilizados em pacientes selecionados, embora a população escolhida nesse estudo tenha sido selecionada por todos já terem tido contato prévio com programas presenciais de reabilitação cardíaca. Em termos de implicações clínicas, esse estudo abre a oportunidade para a implementação de programas de reabilitação cardíaca a distância que possam ser usados como complemento dos programas tradicionais ou que possam ser usados em pessoas provenientes de zonas rurais, longe dos grandes centros hospitalares.

Na fase inicial da pandemia de covid-19, existiu muita divergência sobre o tratamento recomendado a esses pacientes. Apesar de atualmente sabermos que o tratamento com hidroxicloroquina é ineficaz em pessoas infectadas por SARS-CoV-2,^30^ nos primeiros momentos da pandemia essa terapêutica foi considerada uma alternativa de tratamento. Além da eficácia, alguns estudos sugeriram que o tratamento com hidroxicloroquina podia ter efeitos laterais significativos sobre o sistema cardiovascular. Em um estudo publicado em 2022, Hormigo et al.^23^ avaliaram o risco de cardiotoxicidade associado à hidroxicloroquina em uma população de pacientes pediátricos infectados com covid-19, através da monitorização de vários parâmetros do eletrocardiograma (ECG), nomeadamente o intervalo QTc. Nesse estudo, dois dos 14 pacientes precisaram interromper temporariamente o tratamento com hidroxicloroquina devido a um prolongamento do intervalo QTc (> 500 ms), mas todos os pacientes conseguiram completar o tratamento. Esse estudo mostrou, assim, a necessidade de monitorar o risco de cardiotoxicidade da hidroxicloroquina na população pediátrica.

### Doença valvar cardíaca

Em outro artigo, que mostra a importância da medicina translacional, Gavina et al.^14^ fizeram a análise de biópsias do miocárdio obtidas de 56 pacientes submetidos a cirurgia de substituição valvular aórtica por estenose aórtica grave. Em concreto, os autores utilizaram essas biópsias para quantificar a fração de volume de colágeno do miocárdio por histopatologia, com o objetivo de avaliar se a quantidade de fibrose intersticial se associava a pior prognóstico. Os autores demonstraram que a fração de volume de colágeno (sobretudo quando esse valor era superior a 15,4%) era um preditor independente de eventos cardiovasculares e de mortalidade em pacientes com estenose aórtica. Esses resultados podem ter implicações significativas na abordagem e no tratamento dos pacientes com estenose aórtica grave. Por um lado, esses dados demonstram que a presença de uma quantidade significativa de fibrose intersticial se associa a pior prognóstico, o que levanta a hipótese da necessidade de se incorporarem formas não invasivas de avaliação da fibrose do miocárdio (por exemplo, através da ressonância cardíaca com as técnicas de mapeamento T1 ou da avaliação do realce tardio miocárdico) na estratificação de risco dos pacientes com estenose aórtica moderada ou grave. Além disso, esses resultados sugerem a necessidade de se desenvolverem novos fármacos que possam impedir ou atrasar a fibrose miocárdica e, assim, melhorar o prognóstico da estenose aórtica (e outras doenças).

### Endocardite infecciosa

Apesar dos avanços no diagnóstico e tratamento da endocardite infecciosa (EI), essa doença permanece associada a elevada morbimortalidade. Nos últimos anos, alguns estudos têm mostrado uma alteração da epidemiologia da EI, sobretudo em países desenvolvidos. Em um estudo unicêntrico, publicado por Apolinário et al.,^19^ os autores pretenderam analisar a epidemiologia da EI ao longo de um período de 16 anos, de janeiro de 1998 a dezembro de 2013. Nesse estudo, observou-se que, ao longo do tempo, houve uma alteração do perfil de pacientes internados com EI, tendo ocorrido aumento da porcentagem de idosos, maior frequência de doença cardiovascular concomitante e aumento da proporção de pacientes com prótese valvular ou endocardite associada a dispositivos (18% no período anterior a 2008 *versus* 34,6% depois de 2008). Em relação aos microrganismos identificados, o *Staphylococcus aureus* continuou a ser o agente mais frequente, mas, ao longo dos anos, observou-se um aumento das infecções por *Enterococcus* . Nesse estudo, a mortalidade por EI permaneceu muito elevada, com mortalidade intra-hospitalar de 14,5%, mortalidade ao fim de 1 ano de 38% e mortalidade ao fim de 5 anos de 47%, tal como reportado em outras séries hospitalares.^31 , 32^ O estudo da EI é uma área que necessita de mais investigação. Contudo, atendendo à complexidade crescente desses pacientes, é importante discutir também uma melhor organização dos cuidados de saúde dos pacientes com EI, que deve passar pela constituição de centros multidisciplinares de excelência/referência para o tratamento desses pacientes.^33^

### Insuficiência cardíaca

A utilização de dispositivos de assistência ventricular tem tido uma grande evolução nos últimos anos, mas permanece um grande desafio – sobretudo a sua utilização em idade pediátrica. Em um artigo publicado em 2022, Abreu et al. reportaram a experiência clínica de um hospital central no tratamento de 22 casos com dispositivos de assistência ventricular ao longo de vários anos.^21^ Os autores relataram a experiência na utilização de oxigenação por membrana extracorpórea (ECMO, de *extracorporeal membrane oxygenation* ) em pacientes com mediana de idade de 18 meses, de assistência ventricular paracorporal pulsátil em pacientes com mediana de idade de 23 meses e de assistência ventricular paracorporal de fluxo contínuo (AVPFC) em crianças com mediana de idades de 13 anos. As complicações hemorrágicas e tromboembólicas foram as mais frequentes, estando sobretudo relacionadas com a gravidade da doença de base da criança, com o seu peso e com o tipo de dispositivo utilizado. Esse estudo, sendo descritivo, é relevante por mostrar as enormes dificuldades na abordagem desses pacientes, mas também por mostrar a tenacidade e determinação das equipes clínicas na tentativa de melhorar o prognóstico dessas crianças.

### Promoção da saúde cardiovascular

Vários estudos têm mostrado a importância fundamental de ensinar às crianças as medidas de suporte básico de vida (SBV), com objetivo de melhorar os resultados das vítimas de paradas cardiorrespiratórias.^34^ Alguns estudos têm sugerido que é possível que o ensino das medidas de SBV possa ser feito por professores, e não apenas por profissionais de saúde. Em um estudo com desenho quase experimental publicado na Revista Portuguesa de Cardiologia, os investigadores compararam os resultados e os custos do ensino das medidas de SBV realizado por professores (grupo experimental) e por profissionais de saúde (controle) em 362 alunos dos anos 10º a 12º de escolaridade.^22^ Primeiro, nesse estudo observou-se que os resultados foram semelhantes em termos de eficácia da formação na avaliação realizada 2,5 meses após o evento formativo. Contudo, a formação administrada por professores estava associada a custos significativamente inferiores (custos de implementação e manutenção anual, respectivamente, de 4.043 € e 862 € no grupo experimental *versus* 8.561 € e 6.430 € no grupo-controle). Esse estudo é relevante do ponto de vista da sociedade, mostrando que a formação generalizada nas escolas das medidas de SBV pode ser efetiva quando realizada pelos professores estando associada a um custo significativamente menor, o que pode representar um passo fundamental na disseminação do ensino do SBV na sociedade.

### Inteligência artificial na medicina cardiovascular

A inteligência artificial está revolucionando a prática da medicina, sobretudo na análise de dados de saúde, no suporte à decisão clínica e na educação médica. Uma das áreas da medicina cardiovascular que tem tido maior desenvolvimento da inteligência artificial é a análise da imagem médica, nomeadamente da ressonância cardíaca, da tomografia computadorizada cardíaca e da ecocardiografia. Apesar de a utilização de inteligência artificial na cardiologia de intervenção estar menos desenvolvida, o potencial é enorme, permitindo, por exemplo, a identificação anatômica automática das estruturas, a avaliação automática do grau de estenose das artérias coronárias, a melhor identificação das lesões e eventualmente a avaliação funcional das lesões. Em um estudo publicado em 2022, Menezes et al.^20^ mostraram os resultados de um dos primeiros passos necessários para a utilização da inteligência artificial nessa área: a segmentação automática das artérias coronárias. Utilizando os dados de 1.664 imagens, os autores mostraram que é possível o desenvolvimento de modelos de inteligência artificial para essa tarefa com uma boa performance de avaliação após validação por cardiologistas de intervenção.

### Arritmias

A estimulação ventricular direita é a modalidade de estimulação mais utilizada em todo o mundo para correção de distúrbios da condução atrioventricular. Entretanto, esse tipo de estimulação aumenta o risco de fibrilação atrial, pode piorar a classe funcional de insuficiência cardíaca e pode aumentar a necessidade de hospitalização por insuficiência cardíaca em até 20% dos pacientes em 4 anos, especialmente quando a estimulação ventricular se faz necessária > 40% do tempo e em pacientes com disfunção ventricular prévia ao implante.^35^ A estimulação do ramo esquerdo (RE) do sistema His-Purkinje pode evitar desfechos indesejados da estimulação ventricular direita. Forno et al.^11^ avaliaram retrospectivamente os desfechos intraoperatórios, eletrocardiográficos e os dados clínicos do seguimento inicial de 50 pacientes submetidos à estimulação do RE com sucesso no procedimente (n = 52), sendo a maioria do sexo masculino (69,2%) e com mediana de idade de 73,5 anos (65,0-80,0). Os autores concluíram que a estimulação do RE do sistema His-Purkinje é uma técnica segura e exequível, com alta taxa de sucesso, realizada com tempo de procedimento e fluoroscopia baixos, tempo de ativação ventricular esquerdo curto e medidas eletrônicas adequadas.

### Cardiologia de intervenção

Os índices da fisiologia coronária são uma ferramenta essencial na tomada de decisão relacionada a pacientes com doença isquêmica do coração. Porém, foram documentados pontos de cortes diferentes para a relação do ciclo completo de repouso (RFR) que podem ser influenciados pela população e preditores de discordância entre a RFR e a reserva de fluxo fracionado (FFR), dificultando o uso em larga escala dessas técnicas, especialmente na chamda “zona cinzenta”. Fernández-Rodríguez et al.,^12^ empregando dados do estudo RECOPA, analisaram os dados de 156 lesões de 141 pacientes e relacionaram os preditores de discordância em relação à FFR na “zona cinzenta” da RFR (0,86 a 0,92), construindo posteriormente um índice (“RFR ajustada”) que pondera a RFR juntamente com os preditores de discordância. Na sequência, os autores avaliaram a concordância desse índice com a FFR. Eles observaram que os preditores de discordância foram doença renal crônica, cardiopatia isquêmica prévia, lesões não envolvendo a artéria descendente anterior esquerda e síndrome coronariana aguda. Reportaram também que a “RFR ajustada” melhorou a capacidade diagnóstica em comparação com a RFR na “zona cinzenta” (área sob a curva (AUC)-RFR = 0,651 *versus* AUC-“RFR ajustada” = 0,749), com melhora em todos os índices diagnósticos quando foram estabelecidos limiares de corte otimizados (sensibilidade: 59% a 68%; especificidade: 62% a 75%; acurácia diagnóstica: 60% a 71%; razão de verossimilhança positiva: 1,51 a 2,34; razão de verossimilhança negativa: 0,64 a 0,37). Os autores concluíram que a construção de um índice clínico-fisiológico modificado (“RFR ajustada”) incluindo informações sobre a RFR e preditores de discordância melhorou a capacidade diagnóstica na “zona cinzenta”, sendo útil para melhorar a acurácia da RFR e de outros índices da fisiologia coronária.

### Hipertensão arterial pulmonar

Aumentos na resistência da vasculatura pulmonar, causados principalmente pela disfunção endotelial, levam à hipertensão arterial pulmonar (HAP). A resistência da vasculatura pulmonar sobrecarrega o ventrículo direito, resultando em remodelação patológica e disfunção devido a hipertrofia e dilatação. Essa remodelação afeta a dinâmica do ventrículo esquerdo (VE) por causa da interação ventricular direta, especialmente pelo achatamento do septo interventricular.^36^ Soares et al.^13^ estudaram ratos Wistar machos (peso corporal: cerca de 200 g) para investigar se o treinamento físico resistido (TFR) de intensidade baixa a moderada (subir escadas, 55-65% da carga máxima carregada, 5 dias por semana) seria benéfico para funções contráteis do VE e de cardiomiócitos em ratos durante o desenvolvimento de HAP induzida por monocrotalina (MCT). Para testar os efeitos do TFR, os ratos foram divididos entre grupos-controle sedentários (CS, n = 7), hipertensão com sedentarismo (HS, n = 7) e hipertensão com treinamento (HT, n = 7). Os autores observaram que o TFR melhorou a tolerância ao esforço físico (cerca de 55%) e atenuou as disfunções de contratilidade de VE e de cardiomiócitos promovidas pela MCT, preservando a fração de ejeção e o encurtamento fracional, a amplitude do encurtamento e as velocidades de contração e relaxamento nos cardiomiócitos. O TFR também preveniu os aumentos de fibrose e colágeno tipo I no VE causados pela MCT, além de manter as dimensões de miócitos e colágeno tipo III reduzidas por MCT. Os autores sugerem que o treinamento resistido de intensidade baixa a moderada seja testado em pacientes com HAP, embora um longo caminho tenha que ser percorrido até que possamos desenhar um estudo dessa natureza em pacientes com alto grau de complexidade clínica.

## Conclusão

É uma honra renovada este ano para os autores deste artigo poder escrever sobre as melhores publicações científicas no ano de 2022 no Arquivos Brasileiros de Cardiologia e na Revista Portuguesa de Cardiologia.

Novamente, trazemos os achados mais relevantes e seu contexto dentro do cenário da pesquisa em doenças cardiovasculares. Os artigos de 2022 selecionados tiveram tanto temáticas tradicionais como também inovação e tecnologias avançadas. Nas temáticas tradicionais, estiveram presentes estudos investigando e avaliando fatores de risco cardiovascular, promoção de saúde cardiovascular, covid-19 e doença cardiovascular, doença cardíaca isquêmica, doença valvar, insuficiência cardíaca, EI e hipertensão pulmonar. Nas temáticas envolvendo tecnologias avançadas, relatamos o uso da inteligência artificial para a segmentação de artérias coronárias, novas abordagens de estimulação elétrica cardíaca e novos índices para a avaliação da fisiologia do fluxo coronário, além de potenciais usos de técnicas avançadas para avaliação de fibrose miocárdica baseada em dados de estudo translacional na estenose aórtica.

Em 2022, a qualidade científica das publicações nos periódicos científicos mais importantes da Cardiologia em língua portuguesa foi elevadíssima e tornou ainda mais desafiador o trabalho dos autores desta seleção dos 10 melhores artigos. Todos os autores que publicaram em nossas revistas merecem nossos cumprimentos e reverência pela excelência da ciência produzida e pela iniciativa de prestigiar nossas revistas do Brasil e Portugal como veículos para divulgação dos seus dados originais e inovadores. Contamos com as comunidades científicas portuguesa e brasileira para continuar prestigiando nossos periódicos científicos de maior relevância em Cardiologia em 2023. Já antevemos que a pesquisa e o desenvolvimento científico e tecnológico em Cardiologia irão se manter intensos e inovadores no Brasil e em Portugal em 2023.
