# Supplementary material for: Os Melhores Artigos de 2022 nos Arquivos Brasileiros de Cardiologia e na Revista Portuguesa de Cardiologia
Source: Arq Bras Cardiol. 2023 Jul 19;120(7):e20230342. [Article in Portuguese] doi: 10.36660/abc.20230342 (PMC10382155; doi:10.36660/abc.20230342)
Supplement: Translation

# Translation: The Best Articles of 2022 in the *Arquivos Brasileiros de Cardiologia* and *Revista Portuguesa de Cardiologia*

**Authors:** Gláucia Maria Moraes de Oliveira, Ricardo Fontes-Carvalho, Nuno Cardim, Carlos Eduardo Rochitte

**Affiliations:** 1 Brazil Faculdade de Medicina – Universidade Federal do Rio de Janeiro, Rio de Janeiro, RJ – Brazil; 2 Brazil Instituto do Coração Edson Saad – Universidade Federal do Rio de Janeiro, Rio de Janeiro, RJ – Brazil; 3 Portugal Departamento de Cardiologia – Centro Hospitalar de Vila Nova de Gaia/Espinho, Vila Nova de Gaia/Espinho – Portugal; 4 Portugal Cardiovascular Research Center (UniC) - Faculdade de Medicina - Universidade do Porto, Porto – Portugal; 5 Portugal Serviço de Cardiologia Hospital CUF Descobertas, Lisboa – Portugal; 6 Portugal Nova Medical School, Universidade Nova, Lisboa – Portugal; 7 Brazil Instituto do Coração (InCor) – Faculdade de Medicina da Universidade de São Paulo, São Paulo, SP – Brazil; 8 Brazil Hospital do Coração (HCOR), São Paulo, SP – Brazil

**Keywords:** Brazil, Technical Cooperation/trends, Dissemination of Information, Cardiovascular Diseases, Impact Factor

## Introduction

Continuing a recent tradition,^1 , 2^ the *Revista Portuguesa de Cardiologia* and *Arquivos Brasileiros de Cardiologia* are publishing a special article this year with the 10 best original articles published in each journal ( Tables 1 and 2 ).

**Table 1:** – List of the 10 best articles published in *Arquivos Brasileiros de Cardiologia* in 2022

Authors	Article title (link)
Zarife et al. ^4^	Within-Visit Blood Pressure Variability and Cardiovascular Risk in ELSA-Brasil Study Participants (https://abccardiol.org/article/variabilidade-da-pressao-arterial-em-unica-visita-e-risco-cardiovascular-em-participantes-do-elsa-brasil/)
Porto et al. ^5^	Acute Effects of Energy Drink on Autonomic and Cardiovascular Parameters Recovery in Individuals with Different Cardiorespiratory Fitness: A Randomized, Crossover, Double-Blind and Placebo-Controlled Trial (https://abccardiol.org/article/efeitos-agudos-da-bebida-energetica-sobre-parametros-autonomicos-e-cardiovasculares-em-individuos-com-diferentes-capacidades-cardiorrespiratorias-um-ensaio-controlado-randomizado-crossover-e-duplo/)
Jannes et al. ^6^	Screening for Familial Hypercholesterolemia in Small Towns: Experience from 11 Brazilian Towns in the Hipercolbrasil Program (https://abccardiol.org/article/rastreamento-para-hipercolesterolemia-familiar-em-pequenos-municipios-a-experiencia-do-programa-hipercolbrasil-em-11-municipios-brasileiros/)
Kravchychyn et al. ^7^	The Role of ANP Level Response in Interdisciplinary Weight Loss Therapy on Cardiometabolic Risk and Body Composition in Adolescents with Obesity (https://abccardiol.org/article/o-papel-dos-niveis-sericos-de-anp-na-perda-de-peso-risco-cardiometabolico-e-composicao-corporal-de-adolescentes-com-obesidade-submetidos-a-terapia-interdisciplinar/)
Vieira et al. ^8^	Assessment of the Impact of the Implementation of a Pre-Hospital Ambulance System on Acute Myocardial Infarction Mortality in a Developing Country (https://abccardiol.org/article/avaliacao-do-impacto-da-implantacao-de-um-sistema-de-ambulancia-pre-hospitalar-sobre-mortalidade-por-infarto-agudo-do-miocardio-em-um-pais-em-desenvolvimento/)
Bianco et al. ^9^	Pharmaco-invasive Strategy in Myocardial Infarction: Descriptive Analysis, Presentation of Ischemic Symptoms and Mortality Predictors (https://abccardiol.org/article/estrategia-farmaco-invasiva-no-infarto-do-miocardio-analise-descritiva-apresentacao-de-sintomas-isquemicos-e-preditores-de-mortalidade/)
Cerci et al. ^10^	The Impact of COVID-19 on Diagnosis of Heart Disease in Latin America an INCAPS COVID Sub-analysis (https://abccardiol.org/article/o-impacto-da-COVID-19-no-diagnostico-de-doencas-cardiacas-na-america-latina-uma-subanalise-do-incaps-covid/)
Forno et al. ^11^	Left Bundle Branch Pacing of His-Purkinje Conduction System: Initial Experience (https://abccardiol.org/article/estimulacao-do-ramo-esquerdo-do-sistema-his-purkinje-experiencia-inicial/)
Fernández-Rodríguez et al. ^12^	Adjusting RFR by Predictors of Disagreement, “The Adjusted RFR”: An Alternative Methodology to Improve the Diagnostic Capacity of Coronary Indices (https://abccardiol.org/article/ajustando-a-rfr-por-preditores-de-discordancia-a-rfr-ajustada-uma-metodologia-alternativa-para-melhorar-a-capacidade-diagnostica-dos-indices-coronarianos/)
Soares et al. ^13^	Resistance Exercise Training Mitigates Left Ventricular Dysfunctions in Pulmonary Artery Hypertension Model (https://abccardiol.org/article/o-treinamento-fisico-resistido-atenua-as-disfuncoes-ventriculares-esquerdas-em-modelo-de-hipertensao-arterial-pulmonar/)

**Table 2:** – List of the 10 best articles published in the *Revista Portuguesa de Cardiologia* in 2022

Authors	Article title
Gavina et al. ^14^	Prognostic implications of fibrosis in low-risk aortic stenosis patients DOI: 10.1016/j.repc.2021.02.017
Pereira et al. ^15^	Stent-Save a Life international survey on the practice of primary percutaneous coronary intervention during the COVID-19 pandemic DOI: 10.1016/j.repc.2021.04.006.
Pinto et al. ^16^	Digital home-based multidisciplinary cardiac rehabilitation: how to counteract physical inactivity during the COVID-19 pandemic DOI: 10.1016/j.repc.2021.03.012
Almeida et al. ^17^	Prognostic value of brain natriuretic peptide in ST-elevation myocardial infarction patients: a Portuguese registry DOI: 10.1016/j.repc.2020.12.016
Cai et al. ^18^	High expression of SGK1 in thrombosis of acute ST-segment elevation myocardial infarction: based on proteomics analysis of intracoronary thrombosis DOI: 10.1016/j.repc.2021.02.023
Apolinário et al. ^19^	Infective endocarditis: Epidemiology and prognosis DOI: 10.1016/j.repc.2021.02.027
Menezes et al. ^20^	Development of deep learning segmentation models for coronary X-ray angiography: quality assessment by a new global segmentation score and comparison with human performance DOI: 10.1016/j.repc.2022.04.001.
Abreu et al. ^21^	Mechanical circulatory support in children: strategies, challenges and future directions DOI: 10.1016/j.repc.2021.03.011.
Lanzas et al. ^22^	Training program in resuscitation maneuvers delivered by teachers in a school setting: an economic argument DOI: 10.1016/j.repc.2021.02.015
Hormigo et al. ^23^	Protocol-based cardiotoxicity monitoring in hydroxychloroquine medicated COVID-19 pediatric patients DOI: 10.1016/j.repc.2021.01.018

SGK1: serum/glucocorticoid-induced kinase 1.

The studies were selected by the editorial boards of both journals. The process was difficult and complex, since many articles of excellent scientific quality could not be included. However, this illustrates the dynamic nature of cardiovascular research (basic, clinical, and translational) conducted in these Portuguese-speaking countries.

This joint effort to share the best cardiology research in Portuguese-speaking countries is of great importance, especially due to the significant reduction between 1990 and 2019 in age-standardized cardiovascular mortality rates attributable to risk factors, especially in Brazil and Portugal, which have better socioeconomic indices than other Portuguese-speaking countries.^3^ Thus, it is important to share successful experiments from these countries, where the respective journals originate.

### Cardiovascular risk factors

The importance of dietary and metabolic risk factors increased in parallel with reduced smoking rates in Portuguese-speaking countries and the negative correlation between cardiovascular disease (CVD) mortality attributable to risk factors and the sociodemographic index.^3^ However, the leading cause of CVD mortality remained high systolic blood pressure (SBP) in all Portuguese-speaking countries between 1990 and 2019, with greater reductions in Portugal and Brazil.

A 2008-2010 cross-sectional study used baseline data from 14,357 participants of the ELSA-Brasil study with no history of CVD to determine the association between blood pressure variability, measured in a single visit, and cardiovascular risk. Blood pressure variability was quantified using a coefficient of variation of 3 standardized SBP measurements with an oscillometer. Anthropometric measurements and laboratory tests were also performed. Cardiovascular risk was determined with the atherosclerotic cardiovascular disease risk estimator, using multivariate logistic regression analysis and a significance level of 5%. In both sexes, significantly higher cardiovascular risk was associated with elevated blood pressure variability. Significantly higher risk was observed in men than in women across all quartiles, with the greatest difference being in the fourth quartile.^4^ Joint actions, such as developing arterial hypertension treatment guidelines for primary care in Portuguese-speaking countries, especially regarding primary prevention measures, could reduce hypertensive disease outcomes, particularly stroke and acute myocardial infarction (AMI), which are the main causes of mortality in these countries.^24^

In addition to increased SBP, dietary and metabolic risk factors explained the greater variation in CVD burden, which was correlated with the sociodemographic index in Portuguese-speaking countries.^3^ Higher consumption of ultra-processed foods was associated with increased risk of CVD incidence and mortality, suggesting that these foods should be drastically reduced or avoided altogether.^25^ Energy drinks are an ultra-processed food widely consumed to improve aerobic performance, although little is known of their acute effects on cardiovascular physiology. Porto et al.^5^ evaluated the acute effects of a 45-kcal energy drink (250 mL; 11.2 g carbohydrates, 80 mg sodium, 32 mg caffeine, 400 mg taurine, 4.6 mg niacin, 2 mg pantothenic acid, 0.5 mg vitamin B6, 0.4 mg vitamin B12, 240 mg glucuronolactone, and 20 mg inositol) on heart rate variability and cardiovascular recovery after moderate aerobic exercise. The randomized, double-blind, crossover, placebo-controlled study was conducted with a sample of 28 young adults divided into 2 groups according to peak oxygen consumption (VO_2_): (1) high peak VO_2_(peak VO_2_> 52.15 mL/kg/min) and (2) low peak VO_2_(peak VO_2_< 52.15 mL/kg/min). Acute energy drink ingestion had no effect on diastolic or SBP, arterial oxygen saturation by pulse oximetry, or respiratory rate, but it delayed heart rate recovery after exercise in participants with low or high cardiorespiratory capacity. The authors advised individuals with cardiovascular or metabolic diseases to avoid energy drinks prior to exercise.

Diet, elevated fasting blood glucose, elevated low-density lipoprotein cholesterol, and air pollution were among the top five risk factors for CVD in most Portuguese-speaking countries in both 1990 and 2019, including a trend towards an inverse correlation between the sociodemographic index and the percentage of change. Dietary risk, elevated low-density lipoprotein cholesterol, and high SBP, all statistically significant factors, should be tracked.^3^ Elevated low-density lipoprotein cholesterol occurs in familial hypercholesterolemia (FH), an autosomal dominant disease associated with early atherosclerotic CVD. HipercolBrasil is a cascade screening program for FH that has already identified more than 2000 individuals with genetic variants that cause FH through cascade screening of referred index cases, in addition to individuals with hypercholesterolemia and clinical suspicion of FH. A study that performed cascade screening in 11 small Brazilian municipalities suspected of a high prevalence of individuals with FH (previous appearance of the homozygous phenotype in the municipality or regions with a high frequency of AMI) found 105 index cases and 409 first-degree relatives, averaging 4.67 relatives per index case. The authors suggested that specific geographic regions suspected of having a high prevalence of FH warrant a cascade approach to identify clusters of individuals with FH.^6^

Obesity, characterized as a high body mass index (BMI), was the sixth most important risk factor in most Portuguese-speaking countries in both 1990 and 2019.^3^ It should be pointed out that more than 1 billion people in the world are obese (650 million adults, 340 million adolescents, and 39 million children). The World Health Organization estimates that by 2025 the health of approximately 167 million people, both adults and children, will deteriorate due to overweight or obesity.^26^ Kravchychyn et al.^7^ investigated the hypothesis that interdisciplinary clinical weight loss therapy could reduce the prevalence of metabolic syndrome and cardiometabolic risks in obese adolescents and that such improvement would be associated with changes in atrial natriuretic peptide levels. A sample of 73 obese adolescents underwent a 20-week interdisciplinary weight loss program, including exercise, clinical, nutritional, and psychological approaches. Body composition, biochemical analyses and blood pressure were also measured. After the program, volunteers were classified according to increased (n = 31) or reduced plasma levels of atrial natriuretic peptide (n = 19). Significant reductions in body fat, the triglyceride/high-density lipoprotein cholesterol ratio, and metabolic syndrome (from 23% to 6%) only occurred in the group with increased ANP, although both groups had significantly higher fat-free mass and significantly lower body weight, BMI, and waist, neck, and hip circumference.

### Ischemic heart disease

The search for new prognostic markers in people with acute coronary syndrome (ACS) continues. A study by the National ACS Registry of the Portuguese Society of Cardiology, in which dozens of Portuguese centers participated, the authors investigated the prognostic impact of brain natriuretic peptide (BNP) level during hospitalization for ACS with ST-segment elevation.^17^ Data from 1650 patients were evaluated, of whom approximately 21% had elevated BNP values. As expected, those with higher BNP values were older, had more comorbidities, a lower ejection fraction, and more severe CVD. It was also observed that, even after using propensity matching score techniques, a high BNP value (> 400 pg/mL) was an important prognostic marker for both in-hospital and 1-year mortality. This study suggested that BNP measurement is a simple and accessible tool for additional risk stratification in cases of ST-segment elevation ACS.

Thrombosis plays an essential role in the pathophysiology of ACS, and it is important to continue exploring the pathophysiological mechanisms and pathways involved in triggering intracoronary thrombosis. In a study published in the *Revista Portuguesa de Cardiologia* , Cai et al.^18^ analyzed proteomic expression in intracoronary thrombi extracted by aspiration from patients with ST-segment elevation ACS (n = 30). This report is relevant because previous studies in a number of diseases have shown that proteomic analysis can help identify new biomarkers and new therapeutic targets. In this study, serum/glucocorticoid-induced kinase 1 protein expression was significantly higher in thrombus patients than the control group (17.21 [SD, 2.36] vs 4.14 [SD, 1.17%], p < 0.05). Serum/glucocorticoid-induced kinase 1 is an effector of the phosphatidylinositol-3’-kinase signaling pathway and may be a new therapeutic target for atherothrombotic event prevention, although further studies are needed to confirm this hypothesis.

Effective AMI treatment is directly linked to the time that elapses between the event and medical assistance, with approximately half of AMI deaths occurring outside the hospital environment, which highlights the importance of pre-hospital care and the development of evidence-based AMI care systems. Vieira et al.^8^ analyzed the impact of pre-hospital care on general and in-hospital AMI mortality rates and the AMI hospitalization rate in 853 municipalities in Minas Gerais between 2008 to 2016, using hierarchical Poisson regression modeling. Implementation of a Mobile Emergency Care Service was associated with decreases in overall AMI mortality (odds ratio [OR] = 0.967, 95% CI 0.936-0.998) and in-hospital AMI mortality (OR = 0.914, 95% CI 0.845-0.986), but it was not significantly associated with hospitalization (OR = 1.003, 95% CI 0.927-1.083). The authors concluded that these findings reinforce the fundamental role of prehospital care in AMI treatment and that further investment is needed in such services to improve clinical outcomes in low- and middle-income countries.

During the clinical and electrocardiographic diagnostic process for AMI, differences may arise in how symptoms are treated, especially in specific subgroups, such as women and older adults. A study of 2290 patients found that women had a high prevalence of atypical symptoms, a longer time between the symptom onset and treatment seeking, and delay between arrival at the emergency department and fibrinolysis. In-hospital mortality was 5.6%. Hospital mortality rates were higher among women, older adults, and individuals with diabetes mellitus, obesity, chronic kidney disease, or previous strokes. The sex disparity persists for female patients, including delayed recognition of ischemia symptoms and prompt initiation of fibrinolytic therapy, resulting in worse clinical outcomes. The authors pointed out that Killip-Kimball scores, measured during the first medical consultation, accurately predict fatal events, regardless of the clinical presentation of the acute ischemic event, especially in pharmaco-invasive strategies.^9^

### COVID-19 and cardiovascular disease

The SARS-CoV-2 pandemic has had a huge impact on health systems around the world, particularly CVD care. Several 2022 articles showed the impact of COVID-19 on CVD. Although COVID-19’s enormous disruption to CVD care is quite evident, its medium- and long-term impact remains to be seen.

The International Atomic Energy Agency conducted a worldwide survey on changes in cardiac diagnostic volumes due to COVID-19 between March 2019 and March/April 2020. Social distancing data were collected from Google Community Mobility Reports, while COVID-19 incidence data for each country were collected from Our World in Data. The authors analyzed 194 centers that perform cardiac diagnostic procedures in 19 countries in Latin America, finding that, compared to March 2019, the volumes of cardiac diagnostic procedures decreased by 36% in March 2020 and 82% in April 2020. The largest reductions occurred in echocardiographic stress tests (91%), treadmill exercise tests (88%), and computed tomography calcium scores (87%), with small variations between Latin American subregions. Changes in social distancing patterns (p < 0.001) were more strongly associated with these reductions than COVID-19 infection rates (p = 0.003).^10^

Another multicenter article published in the *Portuguese Journal of Cardiology* assessed the impact of COVID-19 on the admission of myocardial infarction patients.^15^ In this study, data from 17 countries participating in the “Stent Save a Life” project were analyzed. In the first 2 months of the pandemic, there was a 27.5% overall reduction in hospital admissions for AMI and a 20% reduction in admissions for ST-segment elevation AMI. It is interesting to note that this reduction was observed in all countries except Egypt and Russia, where the pandemic’s impact came later.

These results are similar to 2 other studies that analyzed the Portuguese situation in greater detail. In the first study, Oliveira et al.^27^ retrospectively evaluated the impact of COVID-19 on two Portuguese centers, finding a 26% reduction in ST-segment elevation AMI cases. There was a trend towards greater system delay times and more mechanical complications, with a consequent increase in patient mortality (1.9% vs 12.1%). These data are very significant and should prompt reflection on the indirect effects of the COVID-19 pandemic,^28^ but they should also oblige health systems to be better prepared for new pandemics. These results are similar to those of another 2022 study^29^ on the impact of COVID-19 on one of the largest hospitals in northern Portugal. There was an overall reduction in hospital admissions due to AMI and an increase in more cases with greater left ventricular dysfunction at the time of discharge (55% vs 39%).

However, apart from its negative effects, the COVID-19 pandemic has also forced health systems to improve patient care through telemedicine. Pinto et al.^16^ analyzed the impact of a distance cardiac rehabilitation program developed during the pandemic that included remote consultations, group exercise sessions, and health and psychological education. A total of 95 CVD patients were included in this program, which resulted in increased physical activity time and reduced levels of sedentary lifestyle. The authors concluded that distance cardiac rehabilitation programs are safe and can be used in selected patients, although their population had previously participated in face-to-face cardiac rehabilitation programs. In terms of clinical implications, this study shows the feasibility of distance cardiac rehabilitation programs as a complement to traditional programs and for use in rural areas, far from hospital centers.

In the initial phase of the pandemic, there was much disagreement about the best treatment for COVID-19 infection. Although we currently know that hydroxychloroquine treatment is ineffective against SARS-CoV-2,^30^ at the beginning of the pandemic it was considered an alternative treatment. In addition to efficacy issues, some studies have suggested that hydroxychloroquine treatment could have significant side effects on the cardiovascular system. In a 2022 study, Hormigo et al.^23^ evaluated the risk of cardiotoxicity associated with hydroxychloroquine use in a population of pediatric patients infected with COVID-19, monitoring several electrocardiogram parameters, principally the QTc interval. Hydroxychloroquine treatment was temporarily discontinued 2 of the 14 patients due to QTc prolongation (> 500 ms), but all patients completed the course of treatment. This study thus showed the need to monitor hydroxychloroquine cardiotoxicity risk in pediatric populations.

### Heart valve disease

In another article on the importance of translational medicine, Gavina et al.^14^ analyzed myocardial biopsies from 56 patients who underwent aortic valve replacement surgery for severe aortic stenosis. Specifically, the myocardial collagen volume fraction was assessed by histopathology to determine whether the quantity of interstitial fibrosis was associated with a worse prognosis. The collagen volume fraction, especially when > 15.4%, was an independent predictor of cardiovascular events and mortality in patients with aortic stenosis. These results may have significant implications for severe aortic stenosis treatment. On the one hand, these data show that a significant amount of interstitial fibrosis is associated with a worse prognosis, which highlights the need to incorporate non-invasive methods of assessing myocardial fibrosis (eg, through cardiac magnetic resonance with T1 mapping techniques or delayed myocardial enhancement) to stratify risk in patients with moderate or severe aortic stenosis. On the other hand, these results suggest that new drugs are needed to prevent or delay myocardial fibrosis and thus improve the prognosis of aortic stenosis (and other diseases).

### Infective endocarditis

Despite advances in the diagnosis and treatment of infective endocarditis (IE), this disease remains associated with high morbidity and mortality. Recent studies have shown a change in the epidemiology of IE, especially in developed countries. In a single-center study, Apolinário et al.^19^ analyzed the epidemiology of IE over 16 years (January 1998 to December 2013), finding that the profile of hospitalized IE patients changed over time, with a higher percentage of older adults, a higher frequency of concomitant CVD, and a higher percentage of patients with prosthetic valves or device-related endocarditis (18% before 2008 vs. 34.6% after 2008). *Staphylococcus aureus* continued to be the most frequent agent, but *Enterococcus* infections increased over the study period. IE mortality remained very high, with in-hospital mortality of 14.5%, 1-year mortality of 38%, and 5-year mortality of 47%; these rates have been corroborated by other hospital series.^31 , 32^ Given the growing complexity of the patient profile, IE requires further investigation. Health care for patients with IE must also be better organized, including the creation of multidisciplinary centers of excellence/reference.^33^

### Heart failure

The use of ventricular assist devices has evolved greatly in recent years, although their use in pediatric patients remains a major challenge. In 2022, Abreu et al. reported a central hospital’s clinical experience with 22 cases requiring ventricular assist devices over several years,^21^ including groups that received an extracorporeal membrane oxygenator (median age: 18 months), a pulsatile paracorporeal ventricular assist device (median age: 23 months), and a paracorporeal continuous flow ventricular assist device (median age: 13 years). Hemorrhagic and thromboembolic complications were the most frequent types, mainly related to underlying disease severity, weight, and device type. This descriptive study is relevant in that it shows the enormous treatment difficulties, as well as the tenacity and determination of the clinical teams who are trying to improve the prognosis of these children.

### Cardiovascular health promotion

Several studies have shown the fundamental importance of teaching children and adolescents basic life support (BLS) to improve results for cardiorespiratory arrest victims.^34^ Some studies have suggested that BLS measures can be taught by schoolteachers, not just health professionals. In a quasi-experimental design study published in the *Revista Portuguesa de Cardiologia* , the investigators compared the costs and results of BLS seminars taught by teachers (experimental group) and health professionals (control) to 362 10th to 12th grade students.^22^ First, the effectiveness results were similar in an assessment 2.5 months after the training event. However, teacher-led training had a significantly lower cost (implementation and annual maintenance costs of EUR 4043 and EUR 862, respectively, in the experimental group vs EUR 8561 and EUR 6430, respectively, in the control group). This shows that general BLS training can be as effective and with a significantly lower cost when performed by schoolteachers, which could be a fundamental step toward broadly disseminating BLS in society.

### Artificial intelligence in cardiovascular medicine

Artificial intelligence is revolutionizing medical practice, particularly health care data analysis, clinical decision support, and medical education. The greatest development of artificial intelligence in cardiovascular medicine has been in medical image analysis, namely cardiac magnetic resonance imaging, cardiac computed tomography, and echocardiography. Although artificial intelligence in interventional cardiology is less developed, the potential is enormous, including automatic identification of anatomical structures, automatic assessment of the degree of coronary artery stenosis, better lesion identification, and even functional lesion assessment. In 2022, Menezes et al.^20^ tested an initial step for artificial intelligence use in this field: automatic segmentation of coronary arteries. Using data from 1664 images, the authors showed that it is possible to develop artificial intelligence models for this task, which received a good performance evaluation after validation by interventional cardiologists.

### Arrhythmia

Right ventricular pacing is the most widely used pacing modality to correct atrioventricular conduction disorders. However, it increases the risk of atrial fibrillation, could worsen heart failure functional class, and could increase hospitalization for heart failure in up to 20% of patients over 4 years, especially when ventricular pacing is required > 40% of the time and in patients with ventricular dysfunction prior to implantation.^35^ Pacing the left bundle branch of the His-Purkinje system can prevent unwanted outcomes from right ventricular pacing. Forno et al.^11^ retrospectively evaluated intraoperative, electrocardiographic, and clinical data from the initial follow-up of 50 patients who underwent a successful left bundle branch pacing procedure (n = 52): mostly men (69.2%) with a median age of 73.5 years (65.0-80.0). The authors concluded that pacing the left bundle branch of the His-Purkinje system is a safe and feasible technique with a high success rate that involves low procedure and fluoroscopy time, short left ventricular activation time, and adequate electronic measurements.

### Interventional cardiology

Coronary physiology indices are an essential tool in decision-making for patients with ischemic heart disease. However, cutoff thresholds for the resting full-cycle ratio (RFR) can vary, perhaps influenced by population characteristics and predictors of discordance between RFR and fractional flow reserve, impeding large-scale use of these techniques, especially in the “grey zone”. Fernández-Rodríguez et al.,^12^ using data from the RECOPA study to analyze 156 lesions in 141 patients, related predictors of discordance to fractional flow reserve in the RFR “grey zone” (0.86 to 0.92), developing an “Adjusted RFR” index that weighs RFR and predictors of discordance. They then evaluated this index’s agreement with fractional flow reserve, finding that the predictors of discordance included chronic kidney disease, previous ischemic heart disease, lesions not involving the left anterior descending artery, and acute coronary syndrome. They also reported that the Adjusted RFR increased diagnostic ability in comparison to the RFR “grey zone” (area under the curve: RFR = 0.651 vs Adjusted RFR = 0.749), improving all diagnostic indices when optimized cut-off thresholds were established (sensitivity: 59% to 68%; specificity: 62% to 75%; diagnostic accuracy: 60% to 71%; positive likelihood ratio: 1.51 to 2.34; negative likelihood: 0.64 to 0.37). They concluded that this modified clinical-physiological index improved diagnostic capacity in the “gray zone”, thus improving the accuracy of RFR and other indices of coronary physiology.

### Pulmonary arterial hypertension

Increased resistance in pulmonary vasculature, mainly due to endothelial dysfunction, leads to pulmonary arterial hypertension. Pulmonary vasculature resistance overloads the right ventricle, resulting in pathological remodeling and dysfunction due to hypertrophy and dilation. This remodeling affects left ventricular dynamics due to direct ventricular interaction, particularly by flattening the interventricular septum.^36^ In male Wistar rats (body weight: ~200 g), Soares et al.^13^ investigated whether low-to-moderate intensity resistance training (stair climbing, 55-65% of the maximum load, 5 days a week) would improve LV contractile and cardiomyocyte contractions during the development of monocrotaline-induced pulmonary hypertension (MCT-PH). To test the effects of resistance training, the rats were divided into the following groups: sedentary controls (n = 7), hypertension + sedentary lifestyle (n = 7) and hypertension + training (n = 7). Resistance training improved physical exertion tolerance (~55%) and attenuated left ventricular and cardiomyocyte contractility dysfunction due to MCT-PH, preserving ejection fraction and fractional shortening, the shortening amplitude, and contraction and relaxation velocities in cardiomyocytes. Resistance training also prevented increases in left ventricular fibrosis and type I collagen due to MCT-PH, in addition to maintaining the myocyte dimensions and type III collagen reduced by MCT-PH. The authors suggested that low-to-moderate intensity resistance training should be tested in patients with pulmonary arterial hypertension, although there is much ground to cover before a study of this nature can be designed in patients with high clinical complexity.

## Conclusions

Once again, it is an honor to report the best scientific publications in the *Arquivos Brasileiros de Cardiologia* and in the *Revista Portuguesa de Cardiologia* . We have described the most relevant findings and their context in CVD research. The selected articles for 2022 involved traditional themes, as well as innovation and advanced technologies. Traditional themes included cardiovascular risk factors, cardiovascular health promotion, COVID-19 and cardiovascular disease, ischemic heart disease, valve disease, heart failure, IE, and pulmonary hypertension. Advanced technology themes included using artificial intelligence in coronary artery segmentation, new approaches to electric cardiac stimulation, and new indices for assessing coronary flow physiology, in addition to advanced techniques in myocardial fibrosis assessment based on data from a translational study on aortic stenosis.

The scientific quality of publications in the most important Portuguese-language cardiology journals was extremely high in 2022, making selection of the 10 best articles a great challenge. All authors who published in our journals deserve our respect for their excellent studies and for choosing our journals as vehicles for disseminating their original and innovative content. We are counting on the continued support of the Portuguese and Brazilian scientific communities in 2023. We can already foresee that cardiology research and technological development will remain intense and innovative in Brazil and Portugal in 2023.
